# Genetic and phenotypic profile of Fabry disease in the population of Vale do Paraiba and Eastern São Paulo

**DOI:** 10.1590/2175-8239-JBN-2022-0107en

**Published:** 2023-02-06

**Authors:** Osvaldo Theodoro da Paz, Rosiane Cássia Teixeira Lacerda, Luis Gustavo Modelli de Andrade

**Affiliations:** 1 Universidade Estadual Paulista, Botucatu, SP, Brasil.

**Keywords:** Fabry Disease, Chronic Renal Failure, lysoGB3, Biomarkers, Doença de Fabry, Insuficiência Renal Crônica, lysoGB3, Biomarcadores

## Abstract

**Introduction:**

Fabry disease (FD) is an inborn error of metabolism characterized by α-galactosidase A deficiency. The primary objective was to evaluate the genetic and phenotypic profile of Fabry disease in hemodialysis.

**Methods:**

Observational cohort study to determine the incidence of genetic variations and phenotypic changes for FD in hemodialysis patients in the Paraiba Valley and Eastern São Paulo. Genetic testing for the GLA gene was performed for men and women over 12 years of age at the hemodialysis clinics between January 2016 and December 2019 as a screening protocol.

**Results:**

The cases came from screening exams of the index case among patients with chronic kidney disease, resulting in 17 families and totaling 82 patients under study. The classification of the most prevalent variant was that of uncertain significance (54%), followed by the pathogenic variant (46%). Five patients in two families were described with two types of variants not previously described in the literature, with pathogenic behavior. Comparing the types of variants, the presence of a pathogenic variant was associated with higher levels of lysoGB3, lower values for alpha-GAL activity and higher frequency of symptoms related to FD.

**Conclusion:**

We characterized an extensive population of patients with FD variants with rich genetic, clinical and biomarker details. We believe that this study can help to better characterize the Brazilian population with FD and the most frequent types of variants.

## RESUMO

**Introdução:**

A doença de Fabry (DF) é um erro inato do metabolismo caracterizado pela deficiência da enzima α-galactosidase A. O objetivo primário foi avaliar o perfil genético e fenotípico da doença de Fabry em hemodiálise.

**Métodos:**

Estudo de coorte observacional para determinar a incidência de variações genéticas e alterações fenotípicas para DF em pacientes em hemodiálise no Vale do Paraíba e Zona Leste de São Paulo. O teste genético para o gene GLA foi realizado para homens e mulheres em todos os pacientes das clínicas de hemodiálise maiores de 12 anos entre janeiro de 2016 a dezembro de 2019 como protocolo de rastreio.

**Resultados:**

Os casos foram provenientes de exames de triagem do caso índice entre pacientes portadores de doença renal crônica, resultando em 17 famílias e totalizando 82 pacientes em estudo. A classificação da variante mais prevalente foi a de significado incerto (54%), seguida da variante patogênica (46%). Foram descritos 5 pacientes em duas famílias com dois tipos de variantes ainda não previamente descritos na literatura com comportamento patogênico. Na comparação entre os tipos de variantes, a presença de variante patogênica foi associada a maiores níveis de lysoGB3, menores valores da atividade da alfa-GAL e maior frequência de sintomas relativos à DF.

**Conclusão:**

Caracterizamos uma extensa população de pacientes com variantes para DF com riqueza de detalhes de genética, clínica e de biomarcadores. Acreditamos que este estudo possa auxiliar na melhor caracterização da população brasileira com DF e nos tipos mais frequentes de variantes.

## Introduction

Fabry disease is an inborn error of metabolism characterized by deficiency of the α-galactosidase A enzyme (α-D-galactoside galactohydrolase); α-gal A^1^ leading to the buildup and deposition of glycosphingolipid (Gb3) in lysosomes^2^. Deficient lysosomal α-galactosidase A (α-gal A) activity results in the progressive buildup of globotriaosylceramide (Gb3 or GL-3) and related glycosphingolipids in the lysosomes of various cell types, including capillary endothelium, kidney, cardiac and nerve cells^3^. Buildup in lysosomes begins in childhood, or even in the fetal stage of development^4^. However, unlike other lysosomal storage diseases^5^, most patients remain clinically asymptomatic during the first years of life. In Fabry disease, it is believed that lysosomal storage and cell dysfunction trigger a cascade of events, such as: impaired energy metabolism, damage to small vessels, oxidative stress and tissue ischemia, culminating in cell death and the development of irreversible cardiac and renalfibrosis^3^. The first clinical symptoms interfere with well-being and performance and appear in childhood, typically between 3 and 10 years of age, and generally occur later in women than in men^6^.

Screening exams contribute to the diagnosis of unsuspected cases when performed in high prevalence populations, such as chronic kidney disease, patients with left ventricular hypertrophy without defined etiology, and stroke of uncertain etiology^7^. However, variants of uncertain meaning and even benign variants that do not correspond to the pathology and are found in the normal population can be identified^8,9^. The publication of the results of the genetic variants with their corresponding phenotype is one of the ways to verify the pathogenicity, contributing to the formation of databases of mutations^10^. In Brazil, numerous screening tests have been performed in patients on hemodialysis, but with few publications of these results^11,12^. It is of fundamental importance to know the genetic profile, the incidence and the results of the specific treatment.

The primary objective was to evaluate the genetic and phenotypic profile for Fabry disease in the population undergoing hemodialysis in the Vale do Paraiba and Eastern São Paulo, extending the analysis to the relatives of the affected cases.

## Materials and Methods

### Study Design

This is an observational cohort study to determine the incidence of genetic variations and phenotypic changes for Fabry disease in patients on hemodialysis in the Paraiba Valley and Eastern São Paulo. The study was approved by the research ethics committee of the Faculdade de Medicina de Botucatu (CAE: 45885721.0.0000.5411) and the patients signed an informed consent form.

### Study Population

Patients undergoing hemodialysis in the Paraiba Valley and Eastern São Paulo were submitted to a screening test for Fabry disease between January 2016 and December 2019, by clinical indication according to the protocol of the centers. As it is a genetic disease, in positive cases the analysis was extended to family members, observing the pattern of inheritance linked to the X chromosome.

### Inclusion/Exclusion Criteria

All patients on renal replacement therapy such as hemodialysis were concordant. In the relatives of patients with a positive genetic test, an inheritance pattern linked to the X chromosome was observed up to the third generation. Patients younger than 12 years old were excluded.

### Protocol for Screening and Evaluation of Positive Cases

In men, alpha-GAL enzymatic activity was measured; and in women, the GLA gene sequencing test was performed. Additionally, in men with alpha-GAL activity lower than the reference value (15.3 µmol/L/h), genetic testing for the GLA gene was performed. The cases of positive genetic analysis were followed up by a doctor specialized in Fabry disease to determine the pathogenicity of the variant through clinical and family analysis and complementary exams. Clinical analyzes were carried out including the search for signs and symptoms of Fabry, such as a history of neuropathic pain and search for angiokeratomas. The patients were submitted to a slit lamp examination to look for verticillata cornea, analysis of renal function by estimating glomerular filtration and proteinuria, evaluation of cardiac function and ventricular mass by echocardiogram. The clinical, biochemical and genetic analyzes were extended to the family members of the index case.

### Definitions

All alpha-GAL enzymatic activity analyzes and genetic tests were performed by filter paper analysis in the CENTOGENE laboratory (AG; Schillingallee 68; 18057 Rostock, Germany). LysoGB3 analysis was performed by liquid chromatography mass spectrometry. The reference value for lyso-GB3 was 1.8 ng/ml. Alpha-GAL analysis was performed using the fluorimetry method. The normal value for alpha-GAL activity was 15.3 μmol/L/h. The GLA gene was analyzed by an amplicon-based next-generation sequencing approach. The amplicons cover the entire coding region and the highly conserved exon-intron splice junctions. We achieved a minimum coverage of 20 times for each amplicon. Missing regions or poor quality regions are completed with classic Sanger sequencing to achieve 100% coverage. The reference sequence was: GLA: *NM_000169.2*.

The variant classification was performed by consulting the Fabry database (http://fabry-database.org/). This analysis resulted in the classification as: benign variant, possibly benign, variant of uncertain meaning, possibly pathogenic and pathogenic according to ACMG recommendations^13^. In unreported cases, additional searches were made in the literature (PubMed) and in the ClinVar database (https://www.ncbi.nlm.nih.gov/clinvar). In the absence of previously reported variants of the GLA gene, these patients were reported according to the characteristics that best defined that variant. Cases were reported as pathogenic due to the variant’s silicic prediction, its population frequency, and the presence of clinical Fabry features.

Basal renal function was estimated using the CKD-EPI formula for patients older than 18 years of age^14^. For patients aged between 12 and 18 years, glomerular filtration was estimated using the Schwartz formula^15^. Proteinuria was performed in 24-hour urine collection.

The definitive diagnosis of Fabry disease was established according to European recommendations^16^ and the Brazilian Consensus on Fabry Disease^17^. We define specific FD symptoms as the presence of angiokeratomas, cornea verticillata or neuropathic pain. Neuropathic pain was defined as pain in the hands and/or feet with onset in childhood or adolescence and/or a course characterized by exacerbations caused by fever, exercise, or heat. The angiokeratoma fulfilled the clinical criteria if it was clustered and present in characteristic areas: bathing trunk area, lips and umbilicus. Cornea verticillata was determined by slit lamp in the absence of amphiphilic drug use (ie, amiodarone, chloroquine).

The geographic locations of the cities where index cases and family members live were obtained using Google maps coordinates (https://www.google.com.br/maps/preview). The population of each municipality was obtained from the IBGE, using data for the year 2021. For the municipality of São Paulo, the sub-region where each family resides (Penha and Grajaú) was considered, adding the populations of these two sub-regions. FD incidence calculations were performed per 100,000 inhabitants.

### Groups

For descriptions of genotypic and phenotypic characteristics, the patients were divided into males and females.

Analyzes were performed on patients grouped by type of variant into two groups: variant of uncertain significance (VUS) and pathogenic.

### Sample Calculation

It is estimated that screening tests were performed in approximately 1,980 patients on hemodialysis during the study period. The estimated incidence of Fabry disease in this population is estimated to be between 0.25 and 0.5%^11,12^. According to Laney et al., for each index case, it is estimated that there are 5 family members with FD^18^. Thus, it is estimated that there are 5 to 10 index cases with a Fabry diagnosis and, extending the analysis to family members, a total of 25 to 50 patients is estimated.

### Statistical Analysis

Numerical data were described as median, 25th and 75th percentiles. Normality was measured using the Shapiro test. Continuous data were presented in number and percentage. Missing data were described in all tables. Sensitivity analyzes were performed, excluding patients with VUS.

For comparisons between genders, the Mann-Whitney test was used for continuous variables, and Fisher’s exact test for categorical variables.

For comparison between the different groups, the Mann-Whitney test was used for continuous variables, and the Fisher’s exact test for categorical variables.

For correlation analysis, we used the Spearman’s correlation test. Correlation coefficients were Spearman’s rho. For clinical symptoms, correlation graphs were made using the network plot, created from a correlation panel in which the most highly correlated variables appeared closer together and are joined by arrows. Arrows are colored by their sign (green for positive and red for negative). The proximity of points is determined using multidimensional clustering.

The graphs were built with the ggplot2 package and statistical analyzes were performed with the R software version 4.0.1.

## Results

**Figure 1.**
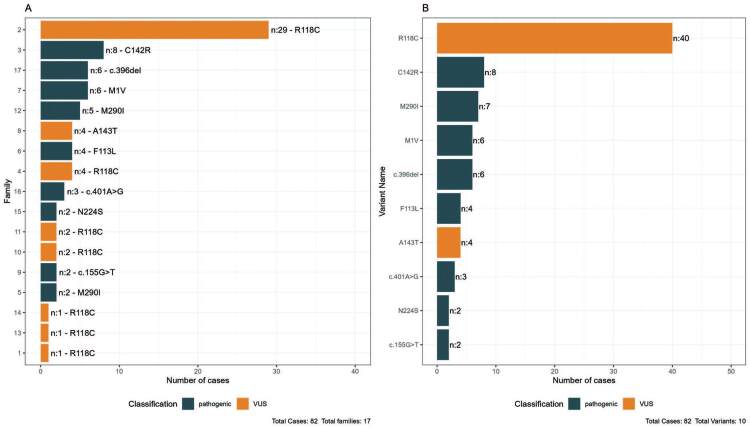
A. Total number of cases and families under study by type of variant in the Fabry study of Vale do Paraiba and Eastern São Paulo. B. Total number of cases by variant type.

There were 94 study cases from 17 different families. Excluding children under 12 years of age, there were 82 cases under analysis. The least numerous family had 1 case and the most numerous, 29 cases. There were 9 families with variants classified as pathogenic and 8 families with variants of uncertain significance (VUS) (Figure 1A). All 82 cases came from the screening of patients on hemodialysis. There were 13 index cases (6 female) from hemodialysis patients and 69 from family screening of the index case.

In total, 10 variants related to the GLA gene have been described. We obtained 8 cases of pathogenic variants (C142R, M290I, c.396del, F113L, N224S, c.401A>G, M1V and c.155G>T) and two variants classified as VUS (R118C, and A143T), (Figure 1B). From the pathogenic group, two variants, not previously described, could be classified as pathogenic (c.401A>G and c.155G>T). The M1V variant reported as VUS can be characterized as pathogenic, due to histological confirmation of the index case.

**Figure 2.**
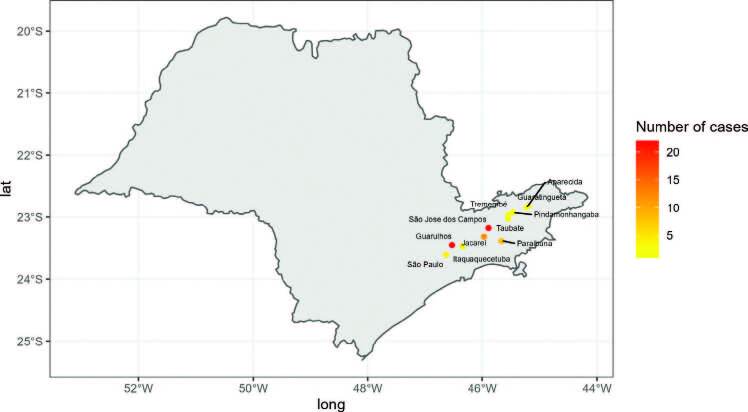
Geographic location of cases and by type of variant in the Fabry study of Vale do Paraiba and Eastern São Paulo.

The geographic location shows the presence of 12 different cities located in the region of Vale do Paraiba and Eastern São Paulo. The cities with the highest number of cases was São José dos Campos (n = 22) and Guarulhos (n = 22). The municipality with the lowest number of cases was Taubaté (n = 1) (Figure 2, Supplementary Table 1).

### Incidence Analysis

For these incidence analyses, only pathogenic variants were considered. The global incidence of Fabry disease presented a frequency of 0.16 cases per 100,000 inhabitants, being lower in Taubaté (0.31 cases/100,000) and higher in Paraibuna (5.46 cases/100,000).

### Descriptive Analysis of the Population

The population under analysis was 82 cases, with 100% of the diagnoses made by screening tests. The median age at diagnosis was 40 (25-52) years, with a prdominance of females (63%). The median age in women was 34 (22-54) years and in men, 45 (39-50) years. Overall, the presence of hypertension was 33%; diabetes, 9%; and median renal function was 92 (69-103) mL/min. Of the total, 54% of the cases were variants of uncertain significance. Symptoms of angiokeratoma and cornea verticillata were present in 9% of the cases. Acroparesthesias were present in 68% of the cases. There was a higher incidence of angiokeratomas in men (21%) compared to 2% in women, p = 0.008. There was a higher incidence of hearing loss in men (25%) compared to zero in women, p < 0.001. Of the total, 54% were being treated with enzyme replacement therapy. Of men, 69% were on ERT, compared to 46% of women, p = 0.038 (Table 1).

**Table 1.** General characteristics, genotype and phenotype of patients with variants for Fabry from Vale do Paraiba and Eastern São Paulo, broken down by sex

Characteristic	Total N = 82	Females N = 52	Males N = 30	p-value
**Age (years)**	40 (25, 52)	34 (22, 54)	45 (39, 50)	0.15
**Race**				>0.9
**White**	79 (96%)	50 (96%)	29 (97%)	
**Black**	3 (3.7%)	2 (3.8%)	1 (3.3%)	
**Smoking**				0.4
**Yes**	7 (8.9%)	3 (6.0%)	4 (14%)	
**No**	72 (91%)	47 (94%)	25 (86%)	
**missing**	3	2	1	
**Hypertension**				0.086
**Yes**	26 (33%)	13 (26%)	13 (45%)	
**No**	53 (67%)	37 (74%)	16 (55%)	
**missing**	3	2	1	
**Diabetes**				>0.9
**Yes**	7 (8.9%)	5 (10%)	2 (6.9%)	
**No**	72 (91%)	45 (90%)	27 (93%)	
**missing**	3	2	1	
**SBP (mmHg)**	120 (110, 132)	120 (110, 133)	120 (110, 130)	0.7
**missing**	10	5	5	
**DBP (mmHg)**	80 (70, 80)	70 (70, 80)	80 (80, 80)	0.056
**missing**	10	5	5	
**eGFR (ml/min)**	92 (69, 103)	93 (74, 109)	78 (64, 102)	0.2
**missing**	22	16	6	
**Proteinuria (mg/24hs)**	22 (0, 85)	45 (0, 78)	1 (0, 100)	0.7
**missing**	24	17	7	
**Genetics**				
**Complete variant**				0.3
c.401A>G p.(Tyr134Cys)	3 (3.7%)	2 (3.8%)	1 (3.3%)	
c.155G>Tp.(Cys52Phe)	2 (2.4%)	1 (1.9%)	1 (3.3%)	
c.1A>Gp.(Mer1?)	6 (7.3%)	2 (3.8%)	4 (13%)	
c.352C>T (p.Arg118Cys)	40 (49%)	26 (50%)	14 (47%)	
c.396del p.(Ile133Phefs*32)	6 (7.3%)	5 (9.6%)	1 (3.3%)	
c.424T>C p.(Cys142Arg)	8 (9.8%)	4 (7.7%)	4 (13%)	
c.427G>Ap.Ala143Thr	4 (4.9%)	3 (5.8%)	1 (3.3%)	
c.671A>G (p. Asn224Ser)	2 (2.4%)	1 (1.9%)	1 (3.3%)	
c.870>A p.M290I	2 (2.4%)	0 (0%)	2 (6.7%)	
c.870G>A p.(Met290Ile)	5 (6.1%)	5 (9.6%)	0 (0%)	
c337T>C p.F113L	4 (4.9%)	3 (5.8%)	1 (3.3%)	
**Variant in short**				0.8
A143T	4 (4.9%)	3 (5.8%)	1 (3.3%)	
c.155G>T	2 (2.4%)	1 (1.9%)	1 (3.3%)	
c.396del	6 (7.3%)	5 (9.6%)	1 (3.3%)	
c.401A>G	3 (3.7%)	2 (3.8%)	1 (3.3%)	
C142R	8 (9.8%)	4 (7.7%)	4 (13%)	
F113L	4 (4.9%)	3 (5.8%)	1 (3.3%)	
M1V	6 (7.3%)	2 (3.8%)	4 (13%)	
M290I	7 (8.5%)	5 (9.6%)	2 (6.7%)	
N224S	2 (2.4%)	1 (1.9%)	1 (3.3%)	
R118C	40 (49%)	26 (50%)	14 (47%)	
**Variant class**				0.7
VUS	44 (54%)	29 (56%)	15 (50%)	
Pathogenic	38 (46%)	23 (44%)	15 (50%)	
**Variant type**				0.4
Deletion	6 (7.3%)	5 (9.6%)	1 (3.3%)	
missense	76 (93%)	47 (90%)	29 (97%)	
**Diagnosis**				
Screening	82 (100%)	52 (100%)	30 (100%)	
**Symptoms**				
**Cornea verticillata**				0.2
**No**	70 (91%)	46 (94%)	24 (86%)	
**Yes**	7 (9.1%)	3 (6.1%)	4 (14%)	
**missing**	5	3	2	
**Angiokeratoma**				0.008
**No**	70 (91%)	48 (98%)	22 (79%)	
**Yes**	7 (9.1%)	1 (2.0%)	6 (21%)	
**missing**	5	3	2	
**Diarrhea**				>0.9
**No**	71 (92%)	45 (92%)	26 (93%)	
**Yes**	6 (7.8%)	4 (8.2%)	2 (7.1%)	
**missing**	5	3	2	
**Orthopnea**				>0.9
**No**	68 (88%)	43 (88%)	25 (89%)	
**Yes**	9 (12%)	6 (12%)	3 (11%)	
**missing**	5	3	2	
**Palpitations**				0.6
**No**	47 (61%)	31 (63%)	16 (57%)	
**Yes**	30 (39%)	18 (37%)	12 (43%)	
**missing**	5	3	2	
**Hypoacusis**				0.3
**No**	74 (96%)	46 (94%)	28 (100%)	
**Yes**	3 (3.9%)	3 (6.1%)	0 (0%)	
**missing**	5	3	2	
**Acroparesthesia**				0.6
**No**	25 (32%)	15 (31%)	10 (36%)	
**Yes**	52 (68%)	34 (69%)	18 (64%)	
**missing**	5	3	2	
**Hypohidrosis**				0.5
**No**	32 (42%)	19 (39%)	13 (46%)	
**Yes**	45 (58%)	30 (61%)	15 (54%)	
**Missing**	5	3	2	
**Headache**				0.2
**No**	40 (52%)	23 (47%)	17 (61%)	
**Yes**	37 (48%)	26 (53%)	11 (39%)	
**Missing**	5	3	2	
**Mood changes**				0.7
**No**	66 (86%)	41 (84%)	25 (89%)	
**Yes**	11 (14%)	8 (16%)	3 (11%)	
**missing**	5	3	2	
**Abdominal pain**				0.7
**No**	53 (69%)	33 (67%)	20 (71%)	
**Yes**	24 (31%)	16 (33%)	8 (29%)	
**missing**	5	3	2	
**Hearing loss**				<0.001
**No**	70 (91%)	49 (100%)	21 (75%)	
**Yes**	7 (9.1%)	0 (0%)	7 (25%)	
**missing**	5	3	2	
**Biomarkers and treatment**				
**LysoGB3 (ng/mL)**	2 (1, 4)	2 (1, 3)	2 (2, 44)	0.014
**missing**	2	1	1	
**Alpha-GAL (mmol/h/L)**	4.05 (2.00, 8.28)	NA (NA. NA)	4.05 (2.00, 8.28)	
**missing**	54	52	2	
**ERT**				0.038
**Yes**	44 (54%)	24 (46%)	20 (69%)	
**No**	25 (31%)	21 (40%)	4 (14%)	
**Requested**	12 (15%)	7 (13%)	5 (17%)	
**missing**	1	0	1	

^1^ Median (IQR); n (%); continuous Mann-Whitney testing; Fisher’s Exact Test for categories. SBP: systolic blood pressure; DBP: diastolic blood pressure; eGFR: estimated glomerular filtration rate; VUS (Variant of uncertain significance); ERT (enzyme replacement therapy).

### Sensitive Descriptive Analysis

We carried out analyzes of the study population, excluding cases of variants of uncertain meaning. After excluding the VUS cases, 38 cases remained under analysis (pathogenic variants). The median age was 41 (27-50) years, with a predominance of females (60%). In women, the median age was 35 (24-53) years and in men, 42 (40-50) years. Overall, the presence of hypertension was 31%; diabetes, 8.3%; and median renal function was 97 (77-106) mL/min. Symptoms of angiokeratoma and cornea verticillata were present in 20% of cases. Angiokeratomas were more frequent in men (40%) compared to women (5%), p = 0.027. Hearing loss was greater in men (27%) compared to women, p = 0.027. Acroparesthesias were present in 77% of the cases. Of the total, 74% were undergoing treatment with enzyme replacement therapy, 93% in men and 61% in women, p = 0.13 (Table 2).

**Table 2.** General characteristics, genotype and phenotype of patients with variants for Fabry from Vale do Paraiba and Eastern São Paulo, excluding the variants of uncertain significance

Characteristic	Overall N = 38	Females N = 23	Males N = 15	p-value
**Age (years)**	41 (27, 50)	35 (24, 53)	42 (40, 50)	0.6
**Race**				>0.9
**White**	36 (95%)	22 (96%)	14 (93%)	
**Black**	2 (5.3%)	1 (4.3%)	1 (6.7%)	
**Smoking**				0.3
**Yes**	4 (11%)	1 (4.8%)	3 (20%)	
**No**	32 (89%)	20 (95%)	12 (80%)	
**missing**	2	2	0	
**Hypertension**				0.5
**Yes**	11 (31%)	5 (24%)	6 (40%)	
**No**	25 (69%)	16 (76%)	9 (60%)	
**missing**	11 (31%)	5 (24%)	6 (40%)	
**Diabetes**				0.5
**Yes**	3 (8.3%)	2 (9.5%)	1 (6.7%)	
**No**	33 (92%)	19 (90%)	14 (93%)	
**missing**	2	2	0	
**SBP (mmHg)**	120 (110, 140)	120 (110, 140)	120 (110, 140)	0.7
**missing**	7	5	2	
**DBP (mmHg)**	80 (70, 80)	75 (70, 80)	80 (70, 80)	0.3
**missing**	7	5	2	
**eGFR (mL/min)**	97 (77, 106)	96 (84, 109)	98 (66, 103)	0.5
**missing**	10	6	4	
**Proteinuria (mg/24hs)**	1 (0, 37)	1 (0, 77)	0 (0, 1)	0.8
**missing**	11	7	4	
**Genetics**				
**Complete variant**				0.2
c. 401A>G p.(Tyr134Cys)	3 (7.9%)	2 (8.7%)	1 (6.7%)	
c.155G>Tp.(Cys52Phe)	2 (5.3%)	1 (4.3%)	1 (6.7%)	
c.1A>Gp.(Mer1?)	6 (16%)	2 (8.7%)	4 (27%)	
c.396del p.(Ile133Phefs*32)	6 (16%)	5 (22%)	1 (6.7%)	
c.424T>C p.(Cys142Arg)	8 (21%)	4 (17%)	4 (27%)	
c.671A>G (p. Asn224Ser)	2 (5.3%)	1 (4.3%)	1 (6.7%)	
c.870>A p.M290I	2 (5.3%)	0 (0%)	2 (13%)	
c.870G>A p.(Met290Ile)	5 (13%)	5 (22%)	0 (0%)	
c337T>C p.F113L	4 (11%)	3 (13%)	1 (6.7%)	
**Variant in short**				>0.9
c.155G>T	2 (5.3%)	1 (4.3%)	1 (6.7%)	
c.396del	6 (16%)	5 (22%)	1 (6.7%)	
c.401A>G	3 (7.9%)	2 (8.7%)	1 (6.7%)	
C142R	8 (21%)	4 (17%)	4 (27%)	
F113L	4 (11%)	3 (13%)	1 (6.7%)	
M1V	6 (16%)	2 (8.7%)	4 (27%)	
M290I	7 (18%)	5 (22%)	2 (13%)	
N224S	2 (5.3%)	1 (4.3%)	1 (6.7%)	
**Variant class**				>0.9
**Pathogenic**	38 (100%)	23 (100%)	15 (100%)	
**Type of variant**				0.4
**Deletion**	6 (16%)	5 (22%)	1 (6.7%)	
**missense**	32 (84%)	18 (78%)	14 (93%)	
**Diagnosis**				
**screening**	38 (100%)	23 (100%)	15 (100%)	
**Symptoms**				
**Cornea verticillata**				0.4
**No**	28 (80%)	17 (85%)	11 (73%)	
**Yes**	7 (20%)	3 (15%)	4 (27%)	
**missing**	3	3	0	
**Angikeratoma**				0.027
**No**	28 (80%)	19 (95%)	9 (60%)	
**Yes**	7 (20%)	1 (5.0%)	6 (40%)	
**missing**	3	3	0	
**Diarrhea**				>0.9
**No**	32 (91%)	18 (90%)	14 (93%)	
**Yes**	3 (8.6%)	2 (10%)	1 (6.7%)	
**missing**	3	3	0	
**Orthopnea**				>0.9
**No**	28 (80%)	16 (80%)	12 (80%)	
**Yes**	7 (20%)	4 (20%)	3 (20%)	
**missing**	3	3	0	
**Palpitations**				>0.9
**No**	19 (54%)	11 (55%)	8 (53%)	
**Yes**	16 (46%)	9 (45%)	7 (47%)	
**missing**	3	3	0	
**Hypoacusis**				>0.9
**No**	34 (97%)	19 (95%)	15 (100%)	
**Yes**	1 (2.9%)	1 (5.0%)	0 (0%)	
**missing**	3	3	0	
**Acroparesthesia**				>0.9
**No**	8 (23%)	5 (25%)	3 (20%)	
**Yes**	27 (77%)	15 (75%)	12 (80%)	
**missing**	3	3	0	
**Hypohidrosis**				>0.9
**No**	9 (26%)	5 (25%)	4 (27%)	
**Yes**	26 (74%)	15 (75%)	11 (73%)	
**missing**	3	3	0	
**Headache**				0.6
**No**	16 (46%)	10 (50%)	6 (40%)	
**Yes**	19 (54%)	10 (50%)	9 (60%)	
**missing**	3	3	0	
**Mood change**				>0.9
**No**	29 (83%)	17 (85%)	12 (80%)	
**Yes**	6 (17%)	3 (15%)	3 (20%)	
**missing**	3	3	0	
**Abdominal pain**				>0.9
**No**	19 (54%)	11 (55%)	8 (53%)	
**Yes**	16 (46%)	9 (45%)	7 (47%)	
**missing**	3	3	0	
**Hearing loss**				0.026
**No**	31 (89%)	20 (100%)	11 (73%)	
**Yes**	4 (11%)	0 (0%)	4 (27%)	
**missing**	3	3	0	
**Biomarkers and treatment**				
**lysoGB3 (ng/mL)**	4 (2. 24)	3 (1. 5)	44 (5. 58)	<0.001
**Alpha-GAL (mmol/h/L)**	2.00 (0.83, 2.00)	NA (NA. NA)	2.00 (0.83, 2.00)	
**missing**	24	23	1	
**ERT**				0.13
**Yes**	28 (74%)	14 (61%)	14 (93%)	
**No**	3 (7.9%)	3 (13%)	0 (0%)	
**Requested**	7 (18%)	6 (26%)	1 (6.7%)	

^1^ Median (IQR); n (%); continuous Mann-Whitney testing; Fisher’s exact test for categories. SBP: systolic blood pressure; DBP: diastolic blood pressure; eGFR: estimated glomerular filtration rate; VUS (variant of uncertain significance); ERT (Enzyme Replacement Therapy).

### Detailed Description of VUS Cases

Most cases reported were variants of uncertain significance (VUS). There were 44 cases, corresponding to 52% of the total.

Cases of R118C were more frequent (n = 40). In this group, the median age at diagnosis was 37 (21-53) years, with 65% of women. There were no cases of symptoms of angiokeratomas or presence of verticillata cornea. There were 55% of acroparesthesia and 7.9% of cases of hearing loss. Median renal function was 78 (60-103) mL/min and proteinuria was 59 (1-87) mg/24hs. The median concentration of lysoGb3 was 1.4 (1.2-1.6) ng/ml. The alpha-GAL activity was 8.2 (6.7 to 8.8) mmol/h/L, which corresponds to 54% activity of a reference value. The index case was a female patient on hemodialysis. Of the total, 12 patients (31% of cases) were being treated with enzyme replacement therapy.

The other VUS group was the A143T variant, with 4 cases. The median age of this group was 40 (36-47) years, with 75% women. Estimated renal function was 91 (73-93) mL/min and proteinuria 60 (30-234) mg/24hs. There were no cases of patients with symptoms of verticillata cornea or the presence of angiokeratomas. Symptoms of acroparesthesia occurred in 100% of the cases. Median lysoGB3 was 1.65 (1.58-1.72) ng/mL, and alpha-GAL activity was 3 (3.1-3.1) mmol/h/L. All cases were on enzyme replacement therapy. The index case was a female patient with eGFR = 54 mL/min, proteinuria of 407 mg/24hs and lysoGB3 of 1.8 ng/mL.

### Analysis Among Groups of Variants

We performed a comparison between the two types of variants (VUS and pathogenic). The characteristics of age, sex, presence of comorbidities and renal function were similar between groups. The presence of deletion was more frequent in pathogenic variants compared to VUS: 16% versus 0%, p = 0.008. Specific symptoms of Fabry disease, such as verticillata corneal angiokeratomas, were more frequent in pathogenic variants compared to VUS: 20% versus 0%, p = 0.003. There was no difference in the presence of acroparesthesia between the groups. LysoGB3 concentration was higher in pathogenic variants 4 (2-24) ng/mL compared to VUS variants 1 (1-2) ng/mL, p < 0.001. Alpha-GAL enzymatic activity was lower in pathogenic variants compared to VUS, p < 0.001. Treatment with enzyme replacement therapy was more frequent in pathogenic variants compared to VUS: 74% versus 37%, p < 0.001 (Supplementary Table 2).

### Correlation Analysis of Clinical Symptoms

**Figure 3.**
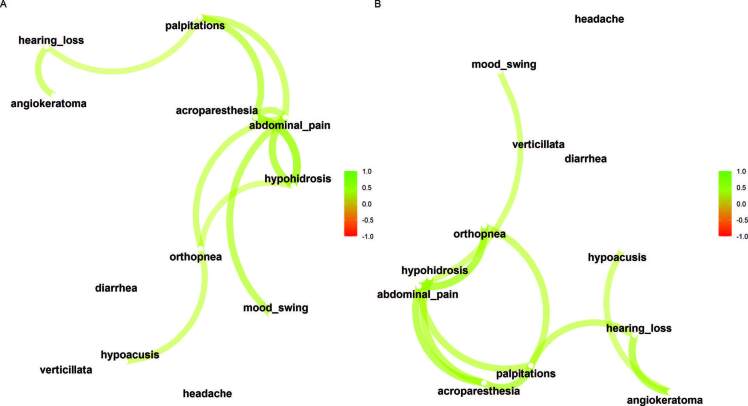
A. Correlation between clinical symptoms in patients with Fabry variants from Vale do Paraiba and Eastern São Paulo (network plot). B. Correlation between clinical symptoms in patients with Fabry variants from Vale do Paraiba and Eastern São Paulo excluding VUS cases (network plot). Correlations are signaled by curves and intensity on a visual color scale. Arrows are colored by their sign (green for positive and red for negative).

We performed a correlation between the clinical symptoms of patients with Fabry variants (n = 82). The diagram graphically represents correlations between symptoms (Figure 3A, Supplementary Figure 1). We see a correlation between hearing loss and angiokeratomas. The symptoms of abdominal pain, acroparesthesia and hypohidrosis were also correlated (Figure 3A, Supplementary Figure 1).

We performed the same correlation analysis between the clinical symptoms of patients with Fabry variants excluding VUS cases (n = 38). The diagram graphically represents correlations between symptoms (Figure 3B, Supplementary Figure 2). We see a correlation between hearing loss, hypoacusis and angiokeratomas. The symptoms of abdominal pain, acroparesthesia and hypohidrosis were also correlated (Figure 3B, Supplementary Figure 2).

### Analysis of Biomarkers and Alpha-GAL Activity

#### Analysis of LysoGB3 in Baseline

**Figure 4.**
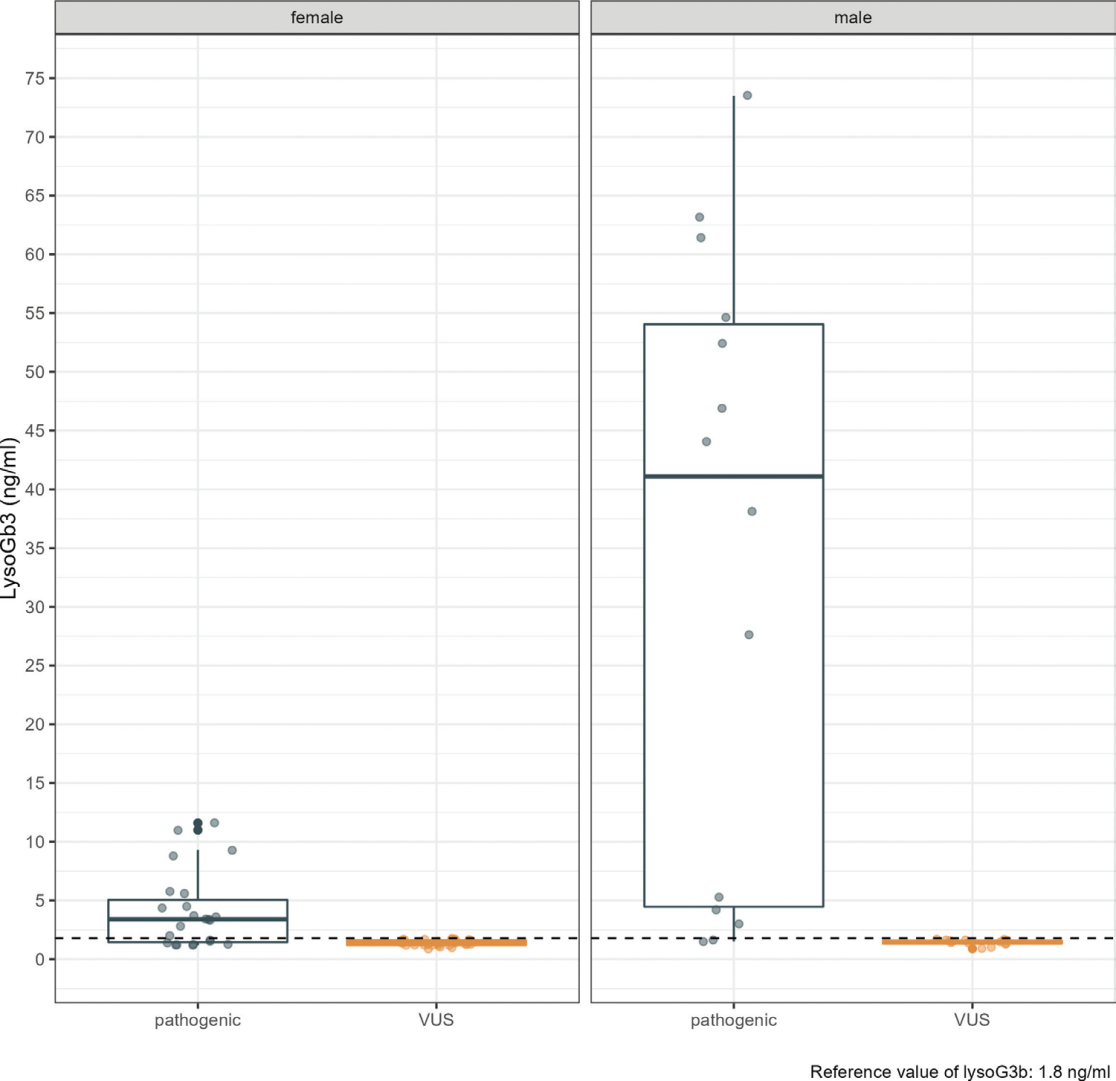
LysoGB3 concentration between the groups of VUS and pathogenic variants in patients with Fabry variants from Vale do Paraiba and Eastern São Paulo. Stratified by sex.

The median concentrations in females for the pathogenic and VUS groups were: 3.40 (1.45-5.05) ng/mL versus 1.40 (1.20-1.63) ng/mL, p < 0.001; respectively. Median concentrations in males for the pathogenic and VUS groups were: 44 (5-58) ng/mL versus 2 (1-2) ng/mL – reference value (less than 1.8 ng/mL); respectively, p < 0.001 (Figure 4).

#### Analysis of Alpha-GAL Activity at Baseline

Analysis of alpha-GAL activity between groups (only in male patients) had median concentrations in the pathogenic variant and VUS groups, respectively: 2.00 (0.83-2.00) versus 7.50 (5 .73-8.78) mmol/h/L – reference value (greater than 15.3 mmol/L/h), p < 0.001 (Figure 6).

**Figure 5.**
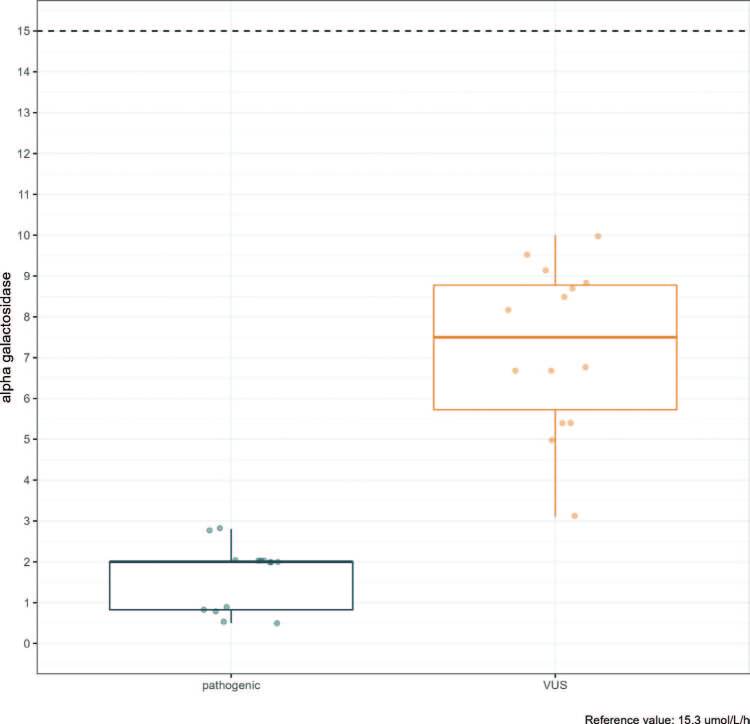
Alpha-GAL concentration between the VUS and pathogenic variants groups in patients with Fabry variants from Vale do Paraiba and Eastern São Paulo.

## Discussion

In this study, we describe the genetic and phenotypic characteristics of 82 patients older than 12 years of age with variants of Fabry disease in the Paraiba Valley and Eastern São Paulo. All cases came from screening exams of the index case among hemodialysis patients, resulting in the identification of 17 families, totaling 82 patients. The most prevalent variant classification was the variant of uncertain significance (n = 44, 54%), followed by the pathogenic variant (n = 38, 46%). Five patients were described in two families with variants not previously described in the literature, presenting pathogenic behavior and included in this category. Additionally, a VUS variant can be classified as pathogenic with biopsy confirmation of the index case (M1V variant). Comparing the types of variants, the presence of a pathogenic variant was associated with higher levels of lysoGB3, lower values of alpha-GAL activity and a higher frequency of symptoms related to Fabry disease (cornea verticillata and angiokeratomas).

### Population Analysis

Data from this study showed a median age at diagnosis of 34 years for women and 45 years for men, with a predominance of females (63%). Excluding patients with VUS, age at diagnosis was 35 years for females and 42 years for males. An analysis of the FOS registry showed the mean age of women at diagnosis to be 37 years and of men to be 34, with a total of 52% of women^19^. In an analysis of the REGISTY cohort in Latin America, the average age of men was 35 years and of women, 37.8 years, with a similar percentage between genders^20^. In the present analysis, we noticed a higher age at diagnosis among men and a higher percentage of diagnosis among women. The higher age at diagnosis can be explained by the difficulty in diagnosing FD and the conditions of the health system. A study of women with FD in Latin America showed a high age at diagnosis, 10 years from the onset of symptoms to diagnosis^20^.

The number of women affected by Fabry disease after the diagnosis of an index case was approximately twice the number of affected men. This number reflects the X-linked inheritance pattern of Fabry disease and suggests that the incidence of Fabry disease in women can be estimated at 1/20.000, compared to the traditional incidence of 1/40,000 in men18. Similarly, a screening study in patients with chronic kidney disease on hemodialysis therapy in Brazil showed an incidence of 66.1% in women, very similar to data from the present study^21^.

### Pathogenic Variants

In this study, 38 variants were classified as pathogenic (c.155G>T, c.396del, c.401A>G, C142R, F113L, M1V, M290I, N224S). Two of these cases (c.401A>G, c.155G>T) not previously described could be classified as pathogenic. The M1V variant described in the literature as VUS can be classified as pathogenic due to the significant reduction in enzymatic activity and the high levels of lyso-GB3. The index case patient underwent a renal biopsy that was compatible with FD.

The presence of classic Fabry disease symptoms was higher in patients with pathogenic variants. The presence of angiokeratomas and cornea verticillata occurred in 20% and hearing loss in 11%. These patients had lower alpha-GAL values and higher lyso-GB3 values compared to patients with VUS. A higher percentage of classic symptoms of angiokeratomas and cornea verticillata was described in men with a classic phenotype compared to the present study^3^. In the present analysis, we found a high percentage of women, which may explain a lower frequency of classic FD symptoms. Possibly these are female patients without the classic FD phenotype. In the view of some authors, the classic phenotype in women is defined as the presence of the pathogenic variant associated with a classic characteristic of FD, such as neuropathic pain, angikeratoma or cornea verticillata^22^. It is also noteworthy that in this group there is a high frequency of neuropathic pain (77%), which could characterize these patients as a classic phenotype. In contrast, neuropathic pain brings greater subjectivity to the evaluation compared to the other two classic signs of FD.

### Variants Not Described

Two families with variants not described in the literature, which in this analysis could be classified as pathogenic, were reported. The first case is from a family with 3 cases of the missense variant c.401A>G. The second case is from a family with 2 cases of the missense variant c.155G>T. The presence of angiokeratomas was 33%; 100% of acroparesthesia; 33% hearing loss; and there were no cases of verticillata cornea. In one of the families we noticed that the measurements of lysoGB3 reached very high values (above 70 ng/mL). Likewise, alpha-GAL activity in men was reduced. We found previously reported as pathogenic associated with the classic phenotype variants similar to those described above. In a variant similar to c.401A>G, a reduction in enzymatic activity has been reported (variant c.401A>C)^23^. In a variant similar to c.155G>T, the presence of a classic FD phenotype with reduced enzymatic activity in the c.155G>A^24^ variant has been reported. Therefore, due to the following characteristics, these variants were classified as pathogenic: in silico prediction of the variant as pathogenic, low population frequency, index case with reduced enzymatic activity and increased lysoGb3 concentration. There was no histological evidence, but renal involvement was attributed to Fabry disease. The patients also presented other symptoms attributed to Fabry (angiokeratomas, acroparesthesia and hearing loss).

### VUS Description

Most of the reported cases fit into cases of variants of uncertain meaning (VUS). There were 44 cases, which corresponds to 53% of the total. As the design of this study was based on a screening test in hemodialysis patients and their families, a greater number of this type of variant is expected. VUS-like variants are more frequent compared to pathogenic variants^7^. Thus, studies that evaluate screening patients show a high percentage of VUS-type variants. The definition of Fabry disease in cases of VUS requires more detailed clinical, laboratory and histological evaluations to characterize the pathogenicity of the variant^9,15,16^.

In the present study, the most frequent variant of the VUS type found was R118C, which occurred in 40 patients, or 49% of the total number of reported cases. The R118C variant may be associated with a reduction in the enzymatic activity of alpha-GAL, and some authors report an association with a late phenotype of Fabry disease, coursing with high levels of lysoGB3^25^. Other authors argue that this variant does not cause the clinical phenotype of Fabry disease^26^. In the present study, we interpreted R118C as a case of late phenotype due to the presence of symptoms (hearing loss, hypoacusis and acroparesthesia); however, a histological and/or biochemical confirmation was not possible.

The other VUS group was the A143T variant (n = 4). These patients had a slight increase in lysoGB3 and symptoms of acroparesthesia. The index case showed evidence of renal involvement. Additionally, a male case showed a 30% reduction in alpha-GAL activity. There are reports of heart disease in the presence of the A143T variant associated with a late phenotype and confirmed by myocardial biopsy showing deposits of GB3^27^. However, other authors advocate that A143T is not pathogenic^28^.

We emphasize that it is essential to confirm the definitive diagnosis of FD before instituting specific therapy. The definitive diagnosis of FD in cases of VUS often requires a renal biopsy^7,15,16^. Performing specific therapy, such as enzyme replacement therapy, without confirming the definitive diagnosis is not appropriate and may generate unnecessary costs and burdens for the government and for the patient.

### Comparison Between Variants

In the pathogenic variants, we found a higher percentage of FD symptoms, such as angiokeratomas, cornea verticillata and neuropathic pain, compared to the VUS variants. These patients have lower values of alpha-GAL activity and higher values of lysoGB3. These data are consistent with the literature, which shows that patients with pathogenic variants are more likely to present the classic phenotype^7^.

### Correlation Between Clinical Symptoms

We performed correlation analysis showing associations between symptoms related to FD. Hearing loss was associated with the presence of angiokeratomas both in the Fabry variant population (rho = 0.37) and in the sensitized analysis excluding VUS cases (rho = 0.49). Angiokeratomas were associated with palpitations, respectively: all Fabry variants (rho = 0.21) and excluding VUS (rho = 0.26). We can also notice a grouping both in all Fabry variants and in the sensitized analysis of the symptoms acroparesthesia, abdominal pain and hypohidrosis.

The frequency of FD symptoms is clearly reported, especially in patients with the classic phenotype^3^; however, the present analysis aggregates the way in which these signs and symptoms are grouped. Thus, the graphic analysis contained in this study can help in understanding how the symptoms are correlated (Figure 3).

## Limitations

This study has some limitations due to its retrospective nature and the occurrence of missing data. We chose to report the number of missing data in the tables. In contrast, there was a small number of missing data, 6% for symptoms, 12% for data on renal function and 4% for comorbidities related to family members of the screening cases. Another limitation of the study was the failure to evaluate the albuminuria that precedes proteinuria in the renal manifestations of FD.

In this analysis, we were unable to report cardiac or neurological assessment data. No histology data are available for VUS cases, especially for the R118C variant and the A143T variant. We cannot, therefore, definitively assign the diagnosis of Fabry disease to the patients with VUS variants in this study.

## Conclusions

This study characterized an extensive cohort of patients with Fabry variants (FD) genetically and phenotypically. Patients with pathogenic variants were associated with a higher occurrence of Fabry symptoms, higher lysoGB3 levels and lower alpha-GAL values. We found a high proportion of variants of uncertain significance (54%) due to the nature of the screening study. The characteristics of patients with VUS that require a more detailed evaluation to confirm the definitive diagnosis of FD have been described. We describe two new, little-reported variants that were considered to be pathogenic. To our knowledge, this is the first Brazilian study characterizing a large population of patients with variants for FD (n = 82) with a wealth of genetic, clinical and biomarker details. We believe that this study can help to better characterize the Brazilian population with FD and the most frequent types of variants found in our country.

## Supplementary Material

The following online material is available for this paper:

Table S1 – Geographic location of cases and by type of variant in the Fabry study of Vale do Paraiba and Eastern São Paulo.

Table S2 – General characteristics, genotype and phenotype of patients with variants for Fabry from Vale do Paraiba and Eastern São Paulo divided by type of variant.

Graph S1 – Correlation between clinical symptoms in the population with Fabry variants in Vale do Paraiba and Eastern São Paulo.

Graph S2 – Correlation between clinical symptoms in patients with Fabry variants from Vale do Paraiba and Eastern São Paulo excluding cases of VUS.
