# Supplementary material for: Genetic and phenotypic profile of Fabry disease in the population of Vale do Paraiba and Eastern São Paulo
Source: J Bras Nefrol. 2023 Feb 6;45(4):424–39. doi: 10.1590/2175-8239-JBN-2022-0107en (PMC10726653; doi:10.1590/2175-8239-JBN-2022-0107en)
Supplement: Supplementary file 1 [file 2175-8239-jbn-2022-0107-s1.pdf]

Braz. J. Nephrol. (J. Bras. Nefrol.)
https://doi.org/10.1590/2175-8239-JBN-2022-0107en

Supplementary Material to "Genetic and phenotypic profile of Fabry disease in the population of Vale do Paraiba and Eastern São Paulo"

Table S1 - Geographic location of the cases by variant type in the Fabry study carried out in the Vale do Paraíba and Eastern São Paulo.

City	Variant	Class variant	N Cases	lat	long
Aparecida	M290I	pathogenic	2	-22.84800	-45.22740
Guaratinguetá	c.396del	pathogenic	3	-22.81620	-45.19350
Guarulhos	A143T	VUS	4	-23.45416	-46.53410
Guarulhos	c.401A>G	pathogenic	3	-23.45416	-46.53410
Guarulhos	M1V	pathogenic	6	-23.45416	-46.53410
Guarulhos	M290I	pathogenic	5	-23.45416	-46.53410
Guarulhos	R118C	VUS	4	-23.45416	-46.53410
Itaquaquecetuba	R118C	VUS	2	-23.48660	-46.34890
Jacareí	R118C	VUS	12	-23.30550	-45.96700
Paraibuna	C142R	pathogenic	8	-23.38140	-45.66260
Pindamonhangaba	F113L	pathogenic	3	-22.92782	-45.45957
São José dos Campos	R118C	VUS	22	-23.17910	-42.88720
São Paulo	c.155G>T	pathogenic	2	-23.61824	-46.63520
São Paulo	N224S	pathogenic	2	-23.61824	-46.63520
Taubaté	F113L	pathogenic	1	-23.03090	-45.54830
Tremembé	c.396del	pathogenic	3	-22.96050	-45.54070
