# Supplementary material for: Genetic and phenotypic profile of Fabry disease in the population of Vale do Paraiba and Eastern São Paulo
Source: J Bras Nefrol. 2023 Feb 6;45(4):424–39. doi: 10.1590/2175-8239-JBN-2022-0107en (PMC10726653; doi:10.1590/2175-8239-JBN-2022-0107en)
Supplement: Supplementary file 2 [file 2175-8239-jbn-2022-0107-s2.pdf]

Braz. J. Nephrol. (J. Bras. Nefrol.)
https://doi.org/10.1590/2175-8239-JBN-2022-0107en

Supplementary Material to "Genetic and phenotypic profile of Fabry disease in the population of Vale do Paraiba and Eastern São Paulo"

Table S2 - General characteristics, genotype and phenotype of patients with Fabry variants in the Vale do Paraíba and Eastern São Paulo broken down by variant type.

	**Pathogenic, N = 38**	**VUS, N = 44**	**p-value**
**Age (years)**	41 (27, 50)	38 (24, 53)	0.8
**Sex**			0.6
**females**	23 (61%)	29 (66%)	
**Males**	15 (39%)	15 (34%)	
**Race**			0.6
**white**	36 (95%)	43 (98%)	
**black**	2 (5.3%)	1 (2.3%)	
**Smoking**			0.7
**yes**	4 (11%)	3 (7.0%)	
**no**	32 (89%)	40 (93%)	
**Missing**	2	1	
**Hypertension**			0.7
**yes**	11 (31%)	15 (35%)	
**no**	25 (69%)	28 (65%)	
**Missing**	2	1	
**Diabetes**			> 0.9
**yes**	3 (8.3%)	4 (9.3%)	
**no**	33 (92%)	39 (91%)	
**Missing**	2	1	
**SBP (mmHg)**	120 (110, 140)	120 (110, 130)	> 0.9
**Missing**	7	3	
**DBP (mmHg)**	80 (70, 80)	80 (70, 80)	0.6
**Missing**	7	3	
**eGFR (mL/min)**	97 (77, 106)	78 (59, 102)	0.11
**Missing**	10	12	
**Proteinuria (mg/24hs)**	1 (0, 37)	60 (1, 88)	0.056
**Missing**	11	13	
**Genetics**			
**Variant type**			0.008
**deletion**	6 (16%)	0 (0%)	
**missense**	32 (84%)	44 (100%)	
**Diagnosis**			
**screening**	38 (100%)	44 (100%)	
**Symptoms**			
**Cornea verticilata**			0.003

	Pathogenic, N = 38	VUS, N = 44	p-value
**no**	28 (80%)	42 (100%)	
**yes**	7 (20%)	0 (0%)	
**Missing**	3	2	
**Angiokeratoma**			0.003
**no**	28 (80%)	42 (100%)	
**yes**	7 (20%)	0 (0%)	
**Missing**	3	2	
**Diarrhea**			> 0.9
**no**	32 (91%)	39 (93%)	
**yes**	3 (8.6%)	3 (7.1%)	
**Missing**	3	2	
**Orthopnea**			0.071
**no**	28 (80%)	40 (95%)	
**yes**	7 (20%)	2 (4.8%)	
**Missing**	3	2	
**Palpitations**			0.3
**no**	19 (54%)	28 (67%)	
**yes**	16 (46%)	14 (33%)	
**Missing**	3	2	
**Hypoacusis**			> 0.9
**no**	34 (97%)	40 (95%)	
**yes**	1 (2.9%)	2 (4.8%)	
**Missing**	3	2	
**Acroparesthesia**			0.10
**no**	8 (23%)	17 (40%)	
**yes**	27 (77%)	25 (60%)	
**Missing**	3	2	
**Hypohidrosis**			0.010
**no**	9 (26%)	23 (55%)	
**yes**	26 (74%)	19 (45%)	
**Missing**	3	2	
**Headache**			0.3
**no**	16 (46%)	24 (57%)	
**yes**	19 (54%)	18 (43%)	
**Missing**	3	2	
**Mood changes**			0.5
**no**	29 (83%)	37 (88%)	
**yes**	6 (17%)	5 (12%)	
**Missing**	3	2	
**Abdominal pain**			0.012
**no**	19 (54%)	34 (81%)	
**yes**	16 (46%)	8 (19%)	
**Missing**	3	2	
**Hearing loss**			0.7
**no**	31 (89%)	39 (93%)	
**yes**	4 (11%)	3 (7.1%)	
**Missing**	3	2	
**Biomarkers and treatment**			
**lysoGB3 (ng/mL)**	4 (2, 24)	1 (1, 2)	< 0.001
**Missing**	0	2	
**Alfa-GAL (mmol/h/L)**	2,00 (0.83, 2.00)	7.50 (5.73, 8.78)	< 0.001
**Missing**	24	30	
**ERT**			< 0.001

	Pathogenic, N = 38	VUS, N = 44	p-value
yes	28 (74%)	16 (37%)	
no	3 (7.9%)	22 (51%)	
Requested	7 (18%)	5 (12%)	
Missing	0	1	

Median (IQR); n (%); Continuous Mann-Whitney test; Fisher's Exact Test for categories

SBP: systolic blood pressure; DBP: diastolic blood pressure; eGFR: estimated renal function; VUS (variant of uncertain significance); ERT (enzyme replacement treatment); Missing: missing data.
