# Supplementary figures and images for: Genetic and phenotypic profile of Fabry disease in the population of Vale do Paraiba and Eastern São Paulo
Source: J Bras Nefrol. 2023 Feb 6;45(4):424–39. doi: 10.1590/2175-8239-JBN-2022-0107en (PMC10726653; doi:10.1590/2175-8239-JBN-2022-0107en)

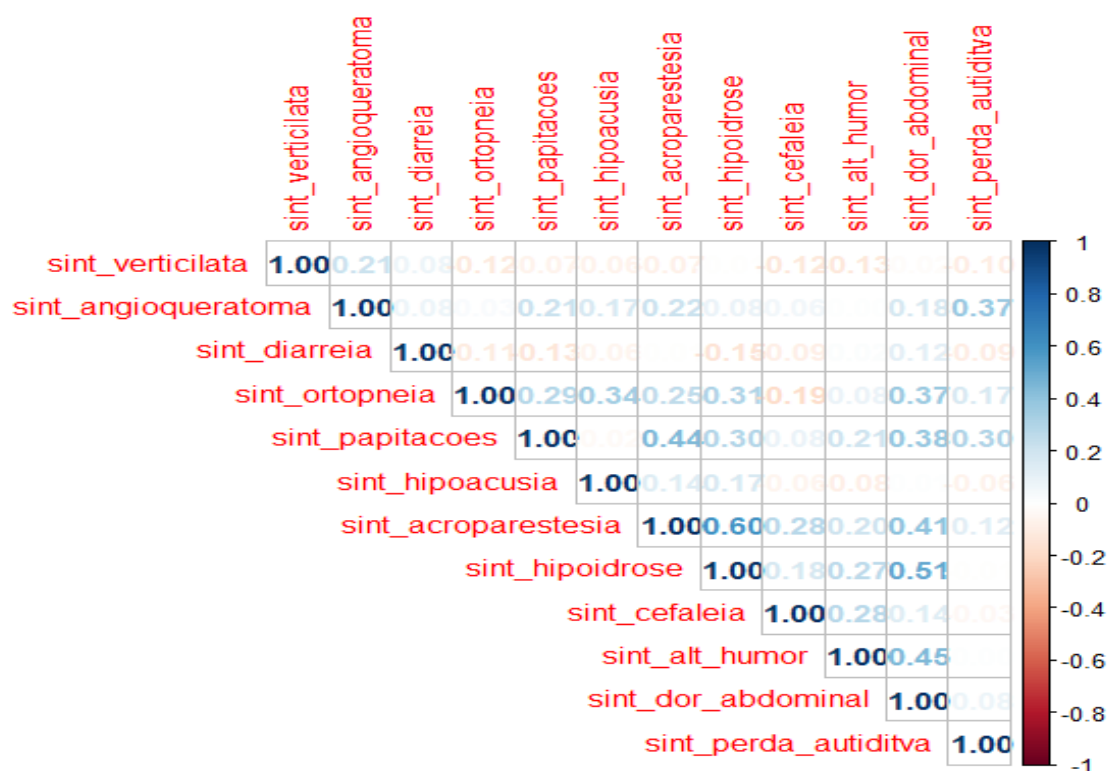

Supplement: Supplementary file 3 [file 2175-8239-jbn-2022-0107-s3.pdf]

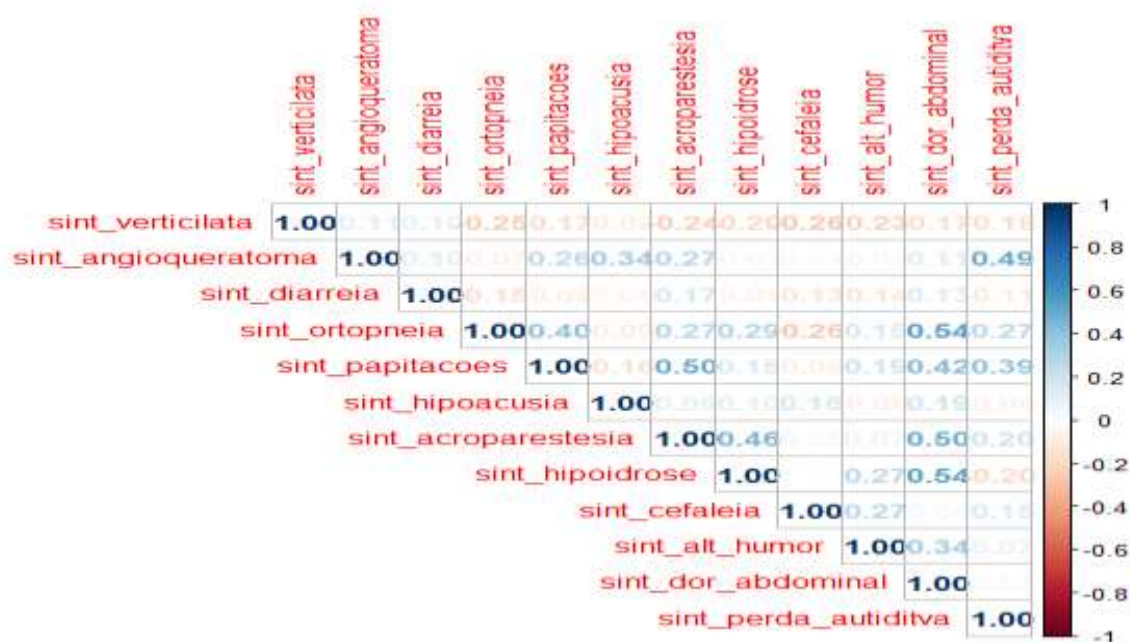

Supplement: Supplementary file 4 [file 2175-8239-jbn-2022-0107-s4.pdf]
